# A Novel CdSe/ZnS Quantum Dots Fluorescence Assay Based on Molecularly Imprinted Sensitive Membranes for Determination of Triazophos Residues in Cabbage and Apple

**DOI:** 10.3389/fchem.2019.00130

**Published:** 2019-03-18

**Authors:** Sihui Hong, Yongxin She, Xiaolin Cao, Miao Wang, Yahui He, Lufei Zheng, Shanshan Wang, A. M. Abd El-Aty, Ahmet Hacimüftüoglu, Mengmeng Yan, Jing Wang

**Affiliations:** ^1^Key Laboratory of Agro-Products Quality and Safety of Chinese Ministry of Agriculture, Institute of Quality Standard and Testing Technology for Agro-Products, Chinese Academy of Agricultural Science, Beijing, China; ^2^College of Life Sciences, Yantai University, Yantai, China; ^3^Department of Pharmacology, Faculty of Veterinary Medicine, Cairo University, Giza, Egypt; ^4^Department of Medical Pharmacology, Medical Faculty, Ataturk University, Erzurum, Turkey

**Keywords:** imprinted array film, triazophos, CdSe/ZnS QDs probe, fluorescence assay, cabbage and apple

## Abstract

In the present study we have developed a direct competitive CdSe/ZnS quantum dot (QD) fluorescence assay based on micro-array-imprinted membranes for the determination of triazophos in cabbage and apple. The imprinted membranes were directly synthesized on the surface of a 96-well plate by thermal polymerization using triadimefon as the dummy template. Under optimal conditions, the assay showed an excellent linear response over the concentration ranges of 0.1–10,000 μg L^−1^ with a good coefficient of determination (*R*^2^= 0.982). The sensitivity (IC_50_) and limit of detection (LOD, expressed as IC_15_) of the developed assay were 3.63 mg L^−1^ and 0.31 μg L^−1^, respectively. The applicability of the developed approach was tested for detecting triazophos in incurred samples. The method showed excellent recoveries (109.6–118.9%) and relative standard deviations (RSDs) between 9.9 and 19.5%. The obtained results correlated well with those obtained by LC-MS/MS (*R*^2^= 0.9995). The competitive assay using CdSe/ZnS QDs as fluorescence-labeled probe showed good sensitivity, steady and fast response, and excellent anti-interference ability compared to conventional fluorescence-quenching methods. Finally, the feasibility of the proposed methodology was successfully applied for detection of triazophos in real samples.

## Introduction

Organophosphate (OPs) insecticides are the most widely used pesticides, including some of the most toxic chemicals used in agriculture (Du et al., 2007). Triazophos (O, O-diethyl-O-(1-phenyl-1H-1, 2, 4-triazol-3-yl) phosphorothioate) has been broadly used as a good substitution of highly toxic OPs to control insect pests on cereals, fruits, vegetables, etc. (Guo et al., 2018). Owing to the high chemical and photochemical stability and long half-life, triazophos may endure in the natural environment (Liu et al., 2015). It was reported that triazophos induced oxidative stress and histopathological changes in rats and may be a potential hazard to human health (Sharma and Sangha, 2014). Since late 2016, the Chinese government has banned the use of triazophos on vegetables. Therefore, it is necessary to develop an effective analytical method for regularly monitoring the pesticide's residual levels in agricultural as well as environment samples (Li H. et al., 2017).

So far, a variety of methods, including gas chromatography (GC) (Xiong and Hu, 2008), gas chromatography coupled with mass spectrometry (GC-MS) (Yang et al., 2011), liquid chromatography coupled with mass spectrometry (LC-MS) (Kasiotis et al., 2014), and enzyme/antibody-based immunoassays (Guo et al., 2009) have been established for sensitive, accurate and specific detection of triazophos. Notably, the high cost and long time for analysis, complex operation, and instrumental size are the major pitfalls for on-site direct detection using GC/LC-MS (Wang et al., 2016; Yang et al., 2018). On the other hand, challenges in antibody production, reagent stability, and the use of laboratory animals may limit the wide utilization of immunoassays (Xie et al., 2010). It is therefore necessary to develop a sensitive, stable, and cheap method for rapid inspection of triazophos to meet the requirements for on-site fast response and control.

Molecular imprinting mimics the principle of the antigen-antibody interaction and is a technique that creates specific recognition sites in synthetic molecularly imprinted polymers (MIPs) as replacements for natural antibodies (Mosbach, 1994). The MIPs have many promising features, such as high chemical stability, good rigidity, specific recognition, low cost, and ease of synthesis (Li L. et al., 2017; Yu et al., 2017). Therefore, MIPs have been widely used in many biomimetic immunosorbent assays, particularly in the biomimetic enzyme-linked immunosorbent assay (Chianella et al., 2013; Shi et al., 2015; Zhang et al., 2018). In most of the aforementioned assays, enzyme-labeled antigen has been used as a probe. However, enzymes are macromolecular substances and enzyme-labeled antigen may cause steric hindrance that could potentially influence the effect of competition and in the end decreases the assay sensitivity (Liu et al., 2018). Recently, QDs have attracted a great deal of attention because of their high sensitivity, superior brightness and photostability, size-dependent emission wavelengths, and narrow emission spectra with broad excitation spectra (Yue et al., 2013; Holzinger et al., 2014; Wang et al., 2018). To find a new marker with smaller size and less complex structure, Liu et al. (2018) developed a direct competitive biomimetic immunosorbent assay (alternative to enzymes) based on the QD label. Because QDs are much smaller than enzymes, they found that QD conjugate was more readily adsorbed by the imprinted film than the enzyme conjugate. Adsorption capacity of MIP film to targets, which depends mostly on sterically restricted sites in films and the recognition ability of hapten labeled with marker, is considered as one of the key factors affecting the sensitivity of biomimetic immunosorbent assay methods based on MIP films. So far, no immunosorbent assay based on MIP film for detecting triazophos residues in ago-products has been reported.

To avoid template leakage, we have selected triadimefon (a structural analog of triazophos) as the dummy template to synthesize the molecularly imprinted array films on the surface of 96-well plates as recognition materials. Next, we have prepared a triazophos hapten conjugated CdSe/ZnS-based quantum dot-labeled probe. Afterwards, a direct competitive CdSe/ZnS quantum dot fluorescence assay based on micro-array-imprinted membranes was developed for determination of triazophos in cabbage and apple. Furthermore, the associated parameters that could affect the performance of the developed method have been optimized and discussed in detail.

## Materials and Methods

### Chemicals and Materials

Triazophos was purchased from Aladdin Chemistry Co. Ltd. (Shanghai, China). Parathion, chlorpyrifos, triadimefon, and methomyl were obtained from Dr. Ehrenstorfer GmbH (Augsburg, Germany). Methacrylic acid (MAA) and trimethylolpropane trimethacrylate (TRIM) were supplied by Alfa Aesar (Massachusetts, USA). The initiator, 2,2-azobisisobutyronitrile (AIBN), was procured from H. V. Chemical Co., Ltd. (Shanghai, China). The triazophos hapten was generously gifted by the Institute of Pesticide and Environmental Toxicology, Zhejiang University, China. QDs (modified with amino-group, emission wavelength (585 nm) were obtained from Wuhan Jiayuan Quantum Dots Co., Ltd. (Wuhan, China). 1-(3-Dimethylaminopropyl)-3-ethylcarbodiimide hydrochloride (EDC) and N-hydroxysuccinate (NHS) were purchased from Sigma-Aldrich (St. Louis, MO, USA). All other chemicals and organic solvents used in experimental works were of analytical grade, unless otherwise specified. The black and flat-bottom 96-well plates were obtained from Corning, Inc. (Corning, NY, USA). Double distilled water (DDW, 18.2 MUcm^−1^) used throughout the experimental works was prepared from a Milli-Q water purification system (Millipore, Bedford, MA, USA).

Phosphate buffered-saline solution (PBS, 10 mM, pH = 7.4), borate buffer saline solution (BBS, 200 mM boric acid root, pH = 8.4), carbonate buffer solution (CBS, 50 mM, pH = 9.6), Tris-HCl buffer (50 mM, pH 8.0), and phosphate buffered saline supplemented with Tween-20 (PBST, consisting of 10 mM PBS (pH 7.4) and 0.05% Tween 20) were used during the experimental works.

### Preparation of Surface Molecularly Imprinted Array Films

Herein, the synthesis of the imprinted film was prepared according to our previous work (Hong et al., 2018). Briefly, triadimefon (2 mmol) was dissolved in 20 mL of ACN, and then MAA (12 mmol) was added. After the mixture was magnetically stirred for 30 min at room temperature, TRIM (6 mmol) and 50 mg of AIBN were sequentially added. The mixture was prepolymerized for 1 h; a total volume of 100 μL assay mixture was added to every well in the black 96-well plate and the reaction was conducted under vacuum at 37°C for 24 h.

**Figure 1:**
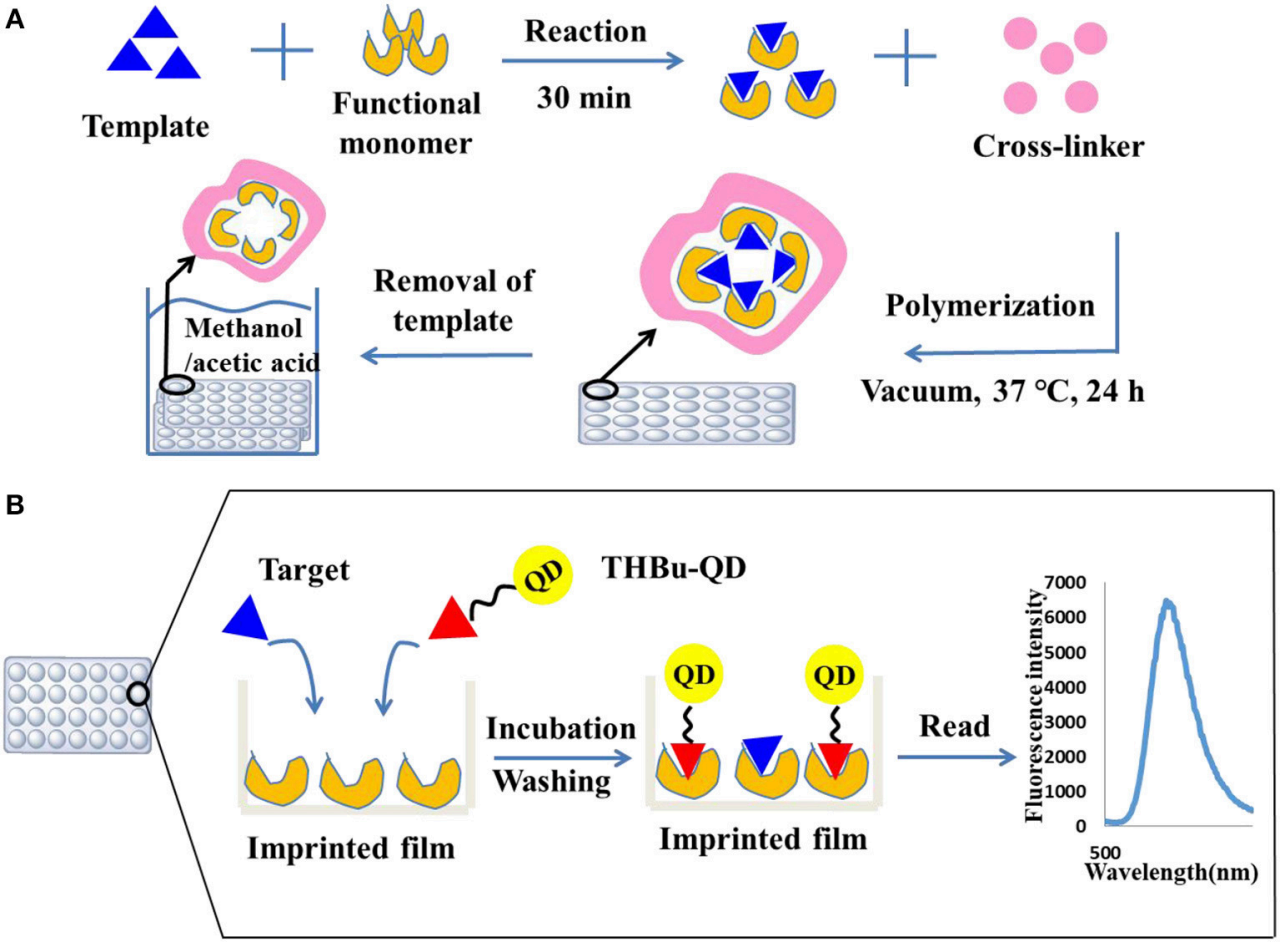
Preparation of the molecularly imprinted film on the surface of the 96-well plate **(A)** and direct competitive BFIA (biomimetic fluorescence immune-sorbent assay) procedure **(B)**.

Unreacted monomers after polymerization were removed by washing the plates three times with DDW and methanol, respectively. The imprinted film was sonicated with eluting solvent (methanol in acetic acid, 7:1, *v/v*) until there was no triadimefon detected in the eluent by HPLC. The plate was then extracted with methanol for 4 h to remove the residual of acetic acid. For comparison, a non-imprinted film was synthesized as stated above without adding triadimefon. The preparation process of the molecularly imprinted film is shown in Figure 1A.

### Synthesis of the CdSe/ZnS-Based Quantum Dot-Labeled Probe for Triazophos

Capping and conjugation of triazophos hapten with CdSe/ZnS QDs was carried out as follows:

#### Preparation of Active Esters

One hundred and sixty microliter of triazophos hapten (15 mmol L^−1^, dissolved in DMF) and 45 μL of NHS (12.3 mg/mL, dissolved in DMF) were added to a centrifuge tube and mixed thoroughly by vortexing for 1 min. Then, 255 μL of EDC (5 mg/mL, dissolved in DMF) was added and the final volume of the solution was adjusted to 1 mL with DMF. The mixture was allowed to react at room temperature over 12 h. The molar ratio of hapten: NHS: EDC was 1:2:3.1 (Wang, 2013).

#### Mark Reaction

Before reaction, 1.5 μL of Tween-20 was added to reduce the reunion of QDs. Then, 50 μL of CdSe/ZnS QDs (dissolved in 340 μL of BBS) and 80 μL of active esters were added to a centrifuge tube and allowed to react at room temperature over 12 h. The molar ratio of active esters: QDs was 480:1 (Wang, 2013).

#### Purification of Markers

The QDs conjugated solution was then dialyzed against PBS (10 mmol L^−1^, pH = 7.4) at room temperature for 1 day (change dialysate every 4 h).

### Direct Competitive CdSe/ZnS QDs Fluorescence Assay Procedure

The direct competitive CdSe/ZnS QDs fluorescence assay procedure was performed using the imprinted array film as recognition elements in the 96-well plate. First, the plate was washed three times with PBST solution. Then, 200 and 100 μL of 10% methanol-BBS (pH = 5) was added to the blank and control wells, respectively. Standard solution or sample extracts were added to the allocated wells (100 μL well^−1^). Next, 100 μL of the CdSe/ZnS QDs-labeled probe was added to all wells except for the blank, and the 96-well plate was shaken for 60 min at room temperature in dark. Following washing with PBST ten times, the fluorescence absorbance was recorded using a Labsystems 96-well plate reader (TECAN, Switzerland) with an excitation wavelength of 368 nm and an emission wavelength of 586 nm. The direct competitive CdSe/ZnS QDs fluorescence assay procedure is shown in Figure 1B. The inhibition rate (IC %) was calculated using Equation (1).

(1) $$\begin{array}{l} IC\left(\%\right)=\left[1-\frac{F_{S}-F_{0}}{F_{c}-F_{0}}\right]\times 100 \end{array}$$

where IC% is the inhibition rate to the target-MIP binding reaction; F_S_ is the average fluorescence value of the standard solution or sample; F_C_ is the average fluorescence value without adding the standard solution; and F_0_ is the average fluorescence value without adding the standard solution and the CdSe/ZnS QDs-labeled probe. Finally, the plates were washed again with methanol and acetic acid (7:1) and then washed with methanol for the next testing.

### Sample Preparation

To evaluate the performance of the developed method (spiked recovery), samples (cabbages and apples) were purchased from a local market. Prior to spiking, LC-MS/MS was used to prove that the samples contained no detectable amount of the tested analyte. The pretreatment process of sample preparation was carried out on the basis of our previous study (Hong et al., 2018).

### HPLC Analysis and LC-MS/MS Validation

The methods of HPLC and LC-MS/MS were conducted in reference to our previous research work (Hong et al., 2018).

## Results and Discussion

### Performance of the Imprinted Film

The FT-IR spectra and SEM of the imprinted film were shown in our previous work (Hong et al., 2018). These results indicate that the imprinted film was synthesized successfully and that the specific binding sites were formed on the surface of the imprinted film. We also found that the imprinted film had a fast adsorption capacity, 74.1% of binding was achieved within 10 min and the equilibrium value was reached at 50 min. It should be noted that the adsorption capacity for the imprinted film was higher than that of the non-imprinted film.

### Characterization of CdSe/ZnS QDs-Labeled Probe of Triazophos

**Figure 2:**
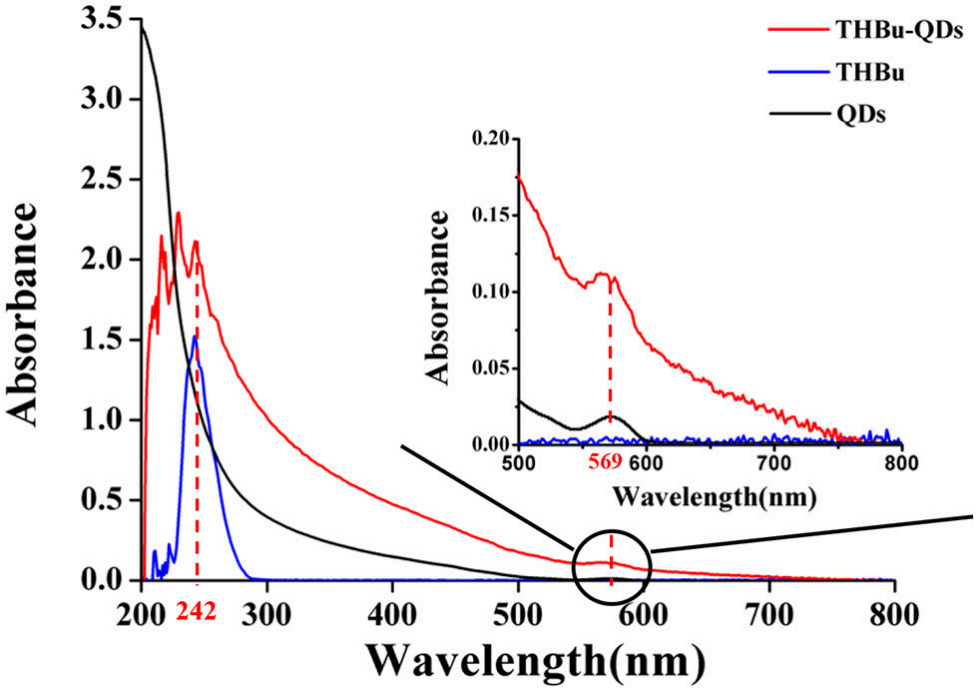
UV-Vis spectra of THBu-QDs (red line), THBu (blue line), and QDs (black line).

The UV-Vis spectra of CdSe/ZnS QDs-labeled probe of triazophos (THBu-QDs, red line), triazophos hapten (THBu, blue line), and CdSe/ZnS QDs (black line) are shown in Figure 2. The peak at 242 and 569 nm were the UV-Vis spectra of THBu and QDs, respectively (Gui, 2007; Unni et al., 2008). The UV-Vis spectrum of THBu-QD has both the UV-Vis spectra of THBu and QDs. This result indicates that the conjugation of triazophos hapten and the CdSe/ZnS QDs was successfully fabricated. Furthermore, the FT-IR spectra of QDs (a), THBu (b), and THBu-QDs (c) are shown in Figure S1. In spectra (a), the IR absorption band around 1,650 cm^−1^ (N–H) indicates the presence of –NH_2_ group. In spectra (b), the peaks at 1,282 cm^−1^ (C-O) and 924 cm^−1^ (O-H, COOH) indicate the presence of the –COOH group. In spectra (c), the peaks at 1,639 cm^−1^ (C=O), and 1,614 cm^−1^ (N–H) represent the -CO-NH- group. These results reveal that the CdSe/ZnS QDs-labeled probe of triazophos was successfully prepared. Additionally, the TEM images of CdSe/ZnS QDs are shown in Figure S2. As shown, the CdSe/ZnS QDs appeared spherical in shape (with diameters ~6 nm) and well-dispersed. The micrograph shows the presence of lattice planes, which confirms the highly crystalline structure of the CdSe/ZnS QDs.

### Optimization of the CdSe/ZnS QDs Fluorescence Assay Conditions

To achieve the optimal sensitivity and precision of the developed method, experimental parameters, including the adding order of the QDs-labeled probe and the standard solution, the QDs-labeled probe concentration, the times of washing the plate, the composition and pH of the working solution, and competition time were optimized and discussed in detail.

The adding order of the QD-labeled probe and the standard solution could affect the CdSe/ZnS QD fluorescence assay. The experimental results showed that the competitive effect of simultaneous addition of the QD-labeled probe and the standard solution was much better than the separate addition of either of them. This finding was in contrast to our previous research (Hong et al., 2018). This might be related to the smaller size of QDs than enzymes. The amino-group of QDs may be easily attached to the carboxyl group of the functional monomer (MAA) when the QD-labeled probe was added first. In turn, it was difficult to replace the QD-labeled probe when the target compound was added thereafter. Thence, the inhibition rate was low.

**Figure 3:**
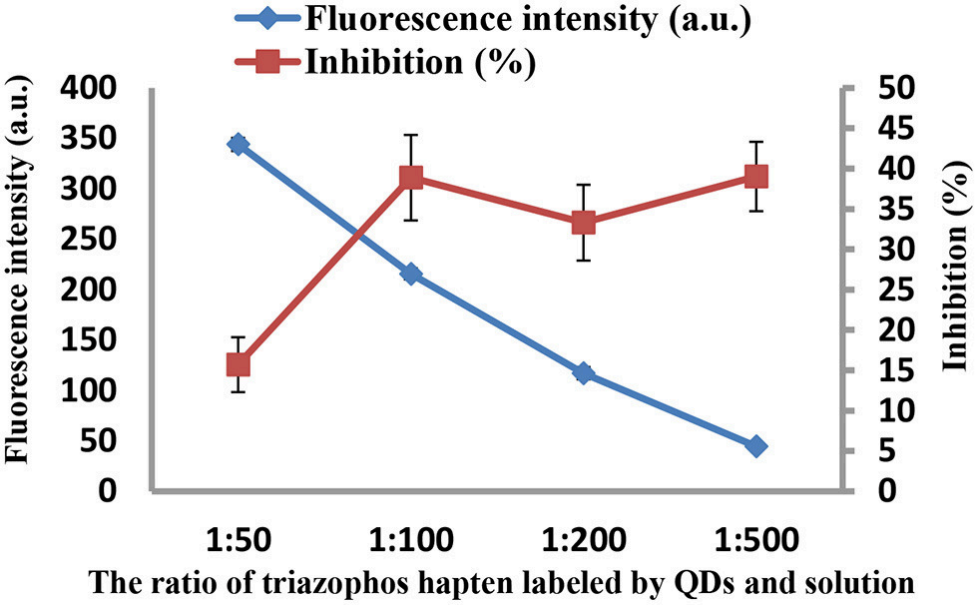
The fluorescence intensity and inhibition of the QD-labeled probe at different dilution ratios.

**Figure 4:**
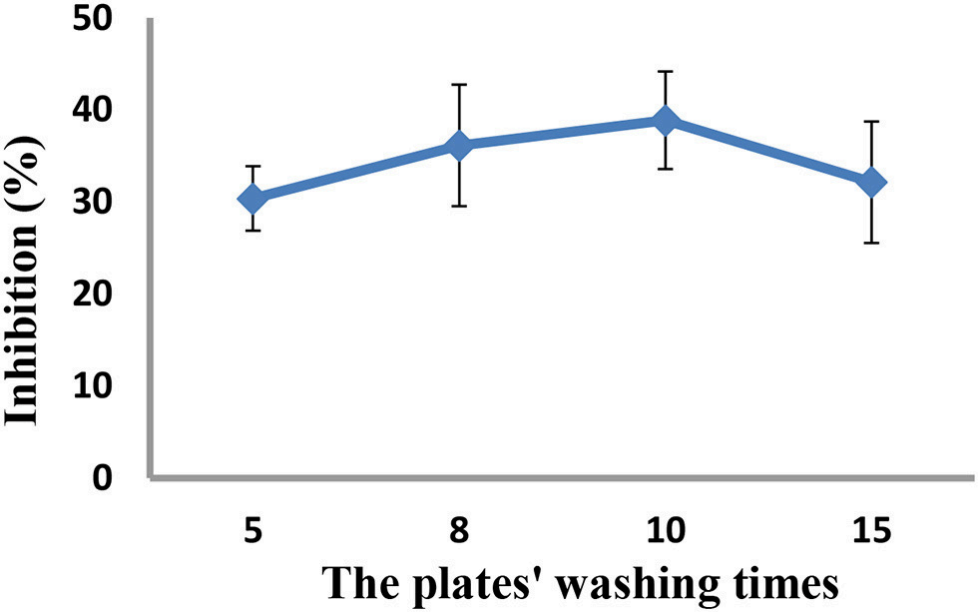
Optimization of the plates' washing times.

As the concentrations of the QD-labeled probe could affect the sensitivity of the developed method, different dilution ratios (1:50, 1:100, 1:200, and 1:500) were therefore tested. As shown in Figure 3, the fluorescence values decreased with decreasing concentrations of the QD-labeled probe, while the inhibition rate was increased. Considering the fluorescence intensity and inhibition rate, the QD-labeled probe was diluted to a ratio of 1:100 before use. Further, the number of washing times for the plate (5, 8, 10, and 15) was tested. As shown in Figure 4, washing ten times had higher inhibition rates compared to others.

The species, concentrations of methanol and pH of the working solution could affect the selectivity and the recognition capability of the imprinted array films, as well as the sensitivity of the method. Herein, different species of working solution (PBS, DDW, BBS, CBS, and TRIS-HCL) were investigated. We obtained a better inhibition ratio and higher sensitivity when BBS was used (Figure S3). To improve the sensitivity of the current method, the concentrations of methanol in BBS solution were optimized (Figure S4): 10% solution of methanol gave the highest sensitivity for triazophos. Working solutions at different pH (5, 6, 7, and 8) were also explored (Figure S5): the highest sensitivity was obtained at pH 5. It has been reported that pH 5–6.5 can alleviate the instability of triazophos, which in turn may increase the sensitivity of the method (Chen and Jia, 2011).

Various competition times (15, 30, 60, 90, and 120 min) were analyzed as well. As shown in Figure S6, the fluorescence efficiency of CdSe/ZnS QDs was high when the competition time was set at 60 min.

### Cross-Reactivity of the CdSe/ZnS QDs Fluorescence Assay

In this experiment, parathion, chlorpyrifos, triadimefon, and methomyl were selected as structural analogs of triazophos and other chemically related compounds to further test the selectivity of the CdSe/ZnS QDs fluorescence assay. The results are shown in Table S1. The cross-reactivity (CR) values were calculated using Equation (2):

(2) $$\begin{array}{l} \text{CR\%}=\left\{\text{I}{\text{C}}_{50}\left(\text{triazophos}\right)/\text{I}{\text{C}}_{50}\left(\text{cross}-\text{reacting compound}\right)\right\}\times 100 \end{array}$$

Relative to triazophos, the CR values of parathion and triadimefon were 6.46 and 3.98%, respectively. This means that the imprinted films have certain specific recognition ability toward compounds with benzene ring, phosphoester bond, or triazole ring. However, the CR values of the four chemically related compounds were <10%. This finding indicates that the imprinted films still have an excellent specificity for triazophos.

### Performance of the CdSe/ZnS QDs Fluorescence Assay

**Figure 5:**
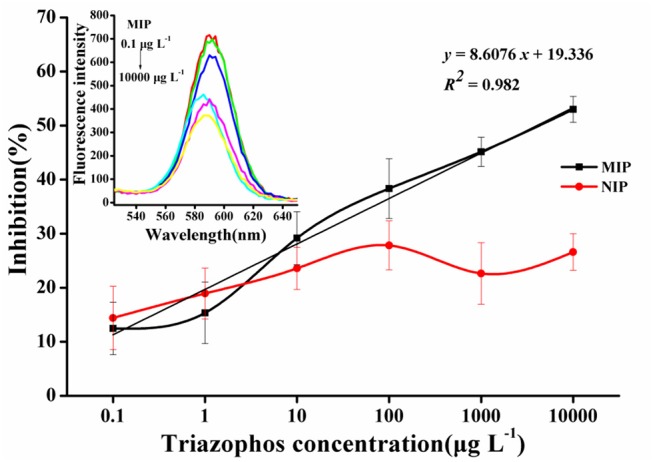
Triazophos BFIA standard curves using the novel imprinted film and the non-imprinted film as an antibody.

The normalized competition curves obtained by the imprinted and the non-imprinted films are shown in Figure 5. The standard curve (*y* = 8.6076*x* + 19.336, *R*^2^ = 0.982, *n* = 3) showed a linear dynamic in the range of 0.1–10,000 μg L^−1^. The sensitivity (IC_50_) was 3.63 mg L^−1^ and the LOD (IC_15_) was 0.31 μg L^−1^. As the concentration of triazophos increases, the gap between MIP and NIP becomes more visible. This finding indicates that the MIP had a better selectivity and adsorption ability for triazophos than the NIP.

Compared to our previous report (Hong et al., 2018), the sensitivity of the CdSe/ZnS QDs fluorescence assay (3.63 mg L^−1^) was lower than that of the BELISA method (428 μg L^−1^). Theoretically, the CdSe/ZnS QDs fluorescence assay should have improved sensitivity. We speculated that it might contribute to a stronger combining ability of CdSe/ZnS QDs probe to the molecularly imprinted membrane than that of enzyme-labeled probe, leading to a reduction in the sensitivity of the method. In general, the developed CdSe/ZnS QDs fluorescence assay exhibits better specific recognition to target molecule and also meets rapid detection requirements of the relevant regulations for triazophos in real samples.

### Preliminary Analysis of Triazophos in Real Samples

The developed method was evaluated by analyzing cabbage and apple samples spiked with trace triazophos at three levels (10, 50, and 500 μg kg^−1^) in triplicates. The results are shown in Table S2. The recovery rates ranged from 114.9 to 118.9% in cabbage and from 109.6 to 117.4% in apple, with the respective RSDs from 14.7 to 19.5% and 9.9 to 17.2%, respectively. Regression analysis was performed to compare the detection results of the developed method and the LC-MS/MS. The obtained correlation coefficient (*R*^2^) was 0.9995. These findings denote that the developed method is reliable for detecting triazophos in agricultural products.

To further demonstrate the applicability of the developed method, samples were collected from different markets located in Beijing (China) and analyzed as stated before. None of the monitored samples tested positive for triazophos. Meanwhile, these samples were verified by LC-MS/MS, and the results were in line with the developed method. This means that the current method can serve as a screening tool for detecting triazophos in practice.

### Comparison to Other Methods

Various detection methods of triazophos were compared in Table S3. Compared with the reported HPLC-MS/MS and antibody-based immunoassays, the current developed method does not need expensive instruments, skilled technicians or hard prepared antibody; additionally, it has low cost and the molecularly imprinted membrane can be reused. Besides, BFIA has a wide linear range and a low LOD, which can meet the needs of analytical testing. Compared with the BELISA method we developed before, BFIA does not require color reaction and termination reaction, which simplified the experimental procedure and shortened the experimental time. With these advantages, BFIA has good potentiality for being applied in the on-site detection of triazophos residues in agriproducts.

## Conclusions

In the present study, a simple, steady, and sensitive fluorescence assay was developed and validated for detecting triazophos using molecularly imprinted array membranes as recognition element and CdSe/ZnS QDs as fluorescence-labeled probe. The developed method was successfully applied for detecting trace levels of triazophos in cabbage and apple, with a lower limit of detection. The developed fluorescence assay method is a feasible and reliable method to monitor triazophos and meets the requirement for detecting its level below the stipulated maximum residue limit (MRL = 0.05 mg kg^−1^) set by China.

## Data Availability

All datasets generated for this study are included in the manuscript and/or the supplementary files.

## Author Contributions

SH carried out experiments and wrote the paper. YS, JW, LZ, and MW designed the experiments, provided useful suggestions, and solved the problems in the experiments. XC and SW provided the experiment tools and helped to synthesis the imprinted film. YH and MY provided the agricultural materials. AA and AH provided useful suggestions to the article and helped to modify the language problem.

### Conflict of Interest Statement

The authors declare that the research was conducted in the absence of any commercial or financial relationships that could be construed as a potential conflict of interest.
