# Supplementary material for: A Novel CdSe/ZnS Quantum Dots Fluorescence Assay Based on Molecularly Imprinted Sensitive Membranes for Determination of Triazophos Residues in Cabbage and Apple
Source: Front Chem. 2019 Mar 18;7:130. doi: 10.3389/fchem.2019.00130 (PMC6432856; doi:10.3389/fchem.2019.00130)
Supplement: Supplementary file 1 [file Data_Sheet_1.docx]

**A novel CdSe/ZnS quantum dots** **fluorescence assay based on molecularly imprinted sensitive membranes for determination of triazophos residues in cabbage and apple**

**Sihui** **Hong^1^, Yongxin She^1^*, Xiaolin Cao^2^, Miao Wang^1^, Yahui** **He^1^, Lufei Zheng^1^,** **Shanshan** **Wang^1^, A. M. Abd EI-Aty^3,4^, Ahmet Hacımüftüoğlu^4^,Mengmeng Yan^1^, and Jing Wang^1^****

^1^*Institute of Quality Standard and Testing Technology for Agro-Products, Chinese Academy of Agricultural Science/Key Laboratory of Agro-Products Quality and Safety of Chinese Ministry of Agriculture, Beijing 100081, P. R. China*

^2^*College of Life Sciences, Yantai University, Yantai 264005, China*

*^3^Department of Pharmacology, Faculty of Veterinary Medicine, Cairo University, 12211-Giza, Egypt*

*^4^Department of Medical Pharmacology, Medical Faculty, Ataturk University, 25240-Erzurum, Turkey*

**Supplementary Materials**

**Fig. S1** FT-IR spectra of QDs (a), THBu (b) and THBu-QDs (c).

**Fig. S2** The TEM images of CdSe/ZnS QDs.

**Fig. S3** BFIA standard curves of triazophos prepared in PBS, DDW, BBS, CBS and TRIS-HCL solution.

**Fig. S4** BFIA standard curves of triazophos in BBS prepared in a series of different concentrations of methanol.

**Fig. S5** BFIA standard curves of triazophos using 10% methanol in BBS at pH 5, 6, 7 and 8.

**Fig. S6** BFIA standard curves of triazophos at various competition time.

**Table S1** Cross-reactivity (CR%) between triazophos and related compounds

**Table S2** Recovery rates of spiked cabbage and apple with triazophos and analyzed by BFIA and LC-MS/MS methods.

**Table S3** Comparison between various detection methods of triazophos.





**Fig. S1** FT-IR spectra of QDs (a), THBu (b) and THBu-QDs (c).


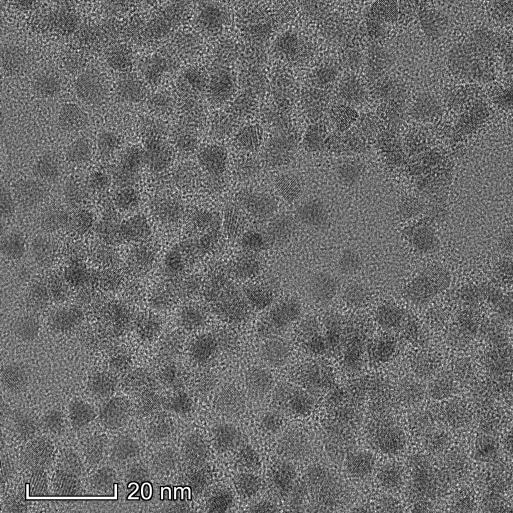

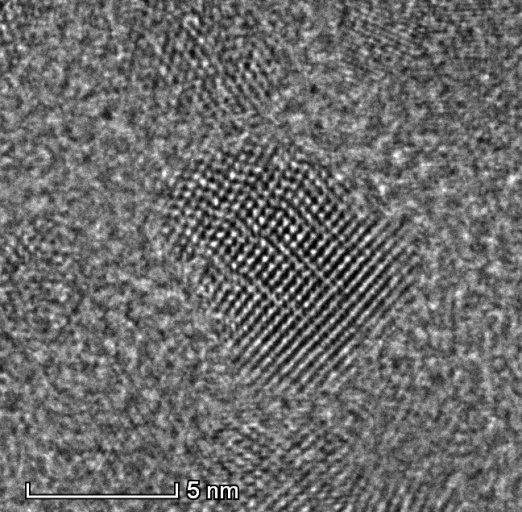


**Fig. S2** The TEM images of CdSe/ZnS QDs.


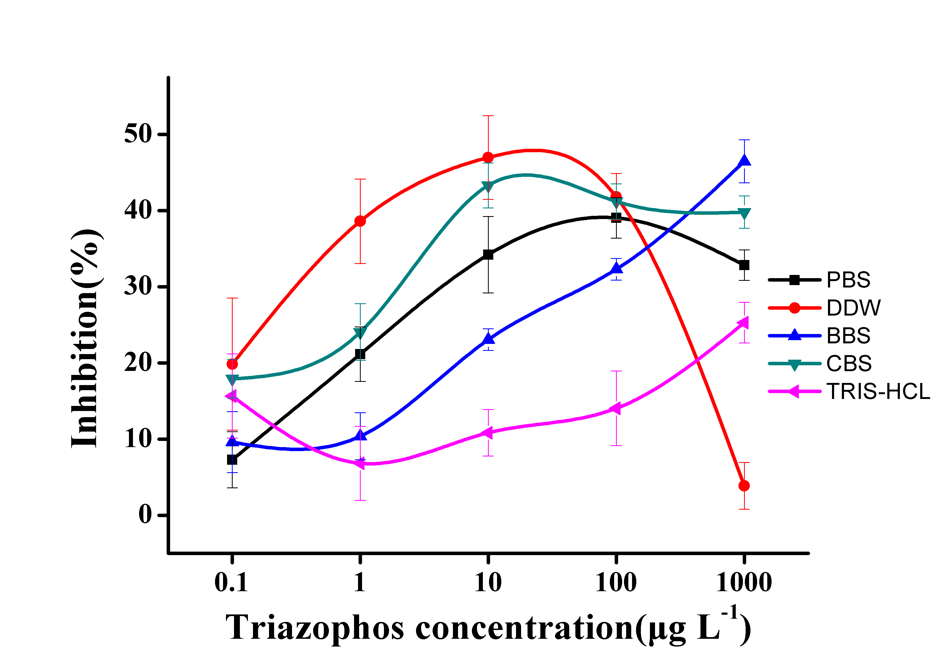


**Fig. S3** BFIA standard curves of triazophos prepared in PBS, DDW, BBS, CBS and TRIS-HCL solution.


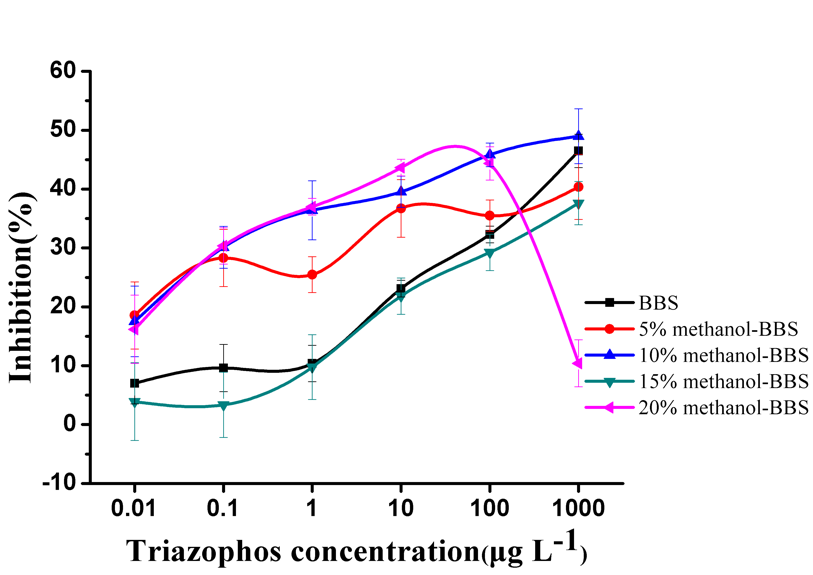


**Fig. S4** BFIA standard curves of triazophos in BBS prepared in a series of different concentrations of methanol.





**Fig. S5** BFIA standard curves of triazophos using 10% methanol in BBS at pH 5, 6, 7 and 8.


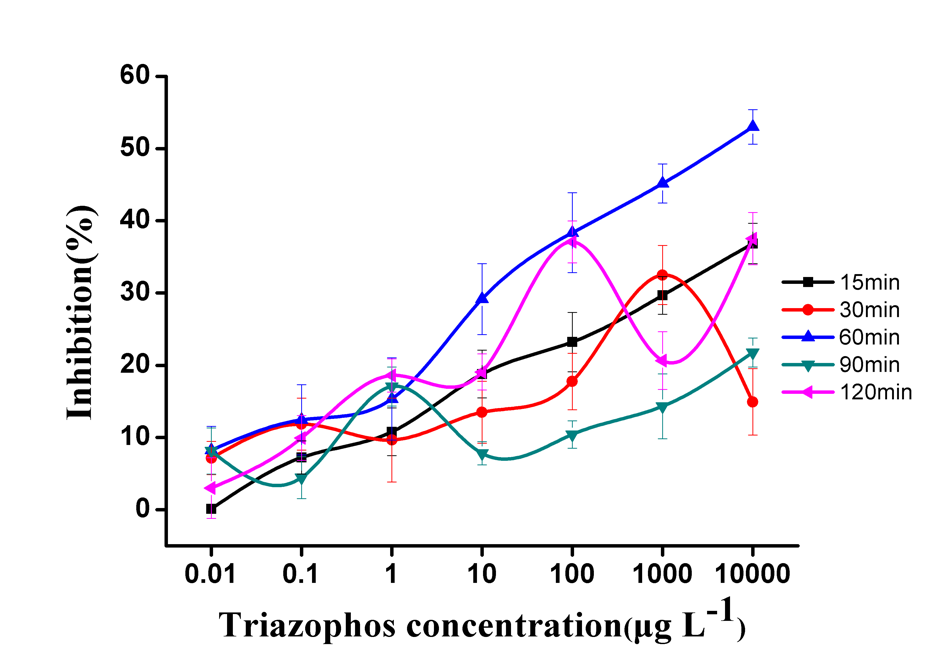


**Fig. S6** BFIA standard curves of triazophos at various competition time.

**Table S1**. Cross-reactivity (CR%) between triazophos and related compounds

| **Analogues** | **Structure** | **IC_50_ (mg L^-1^)** | **CR (%)** |
| --- | --- | --- | --- |
| Triazophos |  | 3.63 | 100 |
| Parathion |  | 56.23 | 6.46 |
| Triadimefon |  | 91.20 | 3.98 |
| Chlorpyrifos |  | >1×10^4^ | <0.03 |
| Methomyl |  | >1×10^4^ | <0.03 |

**Table S2.** Recovery rates of spiked cabbage and apple with triazophos and analyzed by BFIA and LC-MS/MS methods.

| **Sample** | **Spiking level (****μg kg^-1^)** | **Recovery/RSD (%)** | |
| --- | --- | --- | --- |
|  |  | **BFIA** | **LC-MS/MS** |
| Cabbage | 10 | 116.0/14.7 | 85.3/15.0 |
|  | 50 | 114.9/15.6 | 100.9/13.6 |
|  | 500 | 118.9/19.5 | 103.8/7.0 |
| Apple | 10 | 109.6/15.6 | 78.6/2.8 |
|  | 50 | 117.4/17.2 | 105.2/3.5 |
|  | 500 | 115.0/9.9 | 103.7/4.7 |

**Table S3.** Comparison between various detection methods of triazophos

| **Method** | **Sample** | **Linear range (μg L^-1^)** | **LOD (μg L^-1^)** | **Sensitivity**  **(IC_50_, μg L^-1^)** | **Analysis time** | **Cost** | **Reusable**  **Yes/No** | **Ref.** |
| --- | --- | --- | --- | --- | --- | --- | --- | --- |
| HPLC-MS/MS^a^ | Green tea | 2-50 | 0.5 | - | 2 h | High | No | Huang et al., 2019 |
| ELISA^b^ | Water, soil | - | 0.02 | 0.21 | 5 h | High | No | Liang et al., 2007 |
| MIP-CL^c^ | Greengrocery, lettuce, spinach | 1.252-313 | 0.78 | - | 10 min | Low | Yes | Xie et al., 2010 |
| CFBBCIA^d^ | Water, rice, cucumber, apple, cabbage | 0.01-20 | 0.006 | 0.25 | 2.5 h | High | No | Zhang et al., 2018 |
| BELISA^e^ | Cabbage, apple | 0.001-10000 | 0.001 | 428 | 3 h | Low | Yes | Hong et al., 2018 |
| BFIA^f^ | Cabbage, apple | 0.1-10000 | 0.31 | 3630 | 2 h | Low | Yes | This work |

^a^ High performance liquid chromatography-tandem mass spectrometry.

^b^ Enzyme-linked immunosorbent assay.

^c^ Molecular imprinting method for on-line enrichment and chemiluminescent detection.

^d^ Competitive fluorescence bio-barcode immunoassay.

^e^ Biomimetic enzyme-linked immune-sorbent assay.

^f^ Biomimetic fluorescence immune-sorbent assay.
